# Predictive Factors of Canine Malignant Hepatic Diseases with Multifocal Hepatic Lesions Using Clinicopathology, Ultrasonography, and Hepatobiliary Ultrasound Scores

**DOI:** 10.3390/ani14192910

**Published:** 2024-10-09

**Authors:** Aphinan Phosri, Pinkarn Chantawong, Niyada Thitaram, Kidsadagon Pringproa, Atigan Thongtharb

**Affiliations:** 1 Graduate Program in Veterinary Science, Faculty of Veterinary Medicine, Chiang Mai University, Chiang Mai 50100, Thailand; aphinan_phosri@cmu.ac.th; 2 Faculty of Veterinary Medicine, Chiang Mai University, Chiang Mai 50100, Thailand; pinkarn_@hotmail.com (P.C.); niyadathi@gmail.com (N.T.); kdsadagon.p@cmu.ac.th (K.P.); 3 Gastrointestinal and Hepatobiliary Clinic, Small Animal Hospital, Chiang Mai University Animal Hospital, Faculty of Veterinary Medicine, Chiang Mai University, Chiang Mai 50200, Thailand; 4 Surgery Unit, Small Animal Hospital, Chiang Mai University Animal Hospital, Faculty of Veterinary Medicine, Chiang Mai University, Chiang Mai 50200, Thailand; 5 Oncology Clinic, Small Animal Hospital, Chiang Mai University Animal Hospital, Faculty of Veterinary Medicine, Chiang Mai University, Chiang Mai 50200, Thailand; 6 Research Center of Producing and Development of Products and Innovation for Animal Health and Production, Faculty of Veterinary Medicine, Chiang Mai University, Chiang Mai 50100, Thailand

**Keywords:** clinicopathology, dogs, multifocal hepatic lesions, ultrasound hepatobiliary score

## Abstract

**Simple Summary:**

This study investigates predictive factors for distinguishing benign and malignant multifocal hepatic lesions in dogs using ultrasound, hematology, and serum biochemistry. The results show significant differences in hematological and blood chemical parameters, and ultrasonographic parameters. The hepatobiliary ultrasound score and lesion homogeneity show significant differences and high accuracy in predicting malignant multifocal liver lesions, making them effective parameters for screening in dogs.

**Abstract:**

Multifocal hepatic lesions in dogs arise from various benign and malignant liver diseases. Diagnosing these lesions is challenging because clinical signs, hematological data, and serum biochemistry are not definitive indicators. Ultrasound is utilized as a diagnostic imaging tool to evaluate liver parenchyma and detect hepatic lesions. This study aims to investigate the predictive factors that differentiate between benign and malignant multifocal hepatic lesions by examining ultrasound characteristics, blood tests, and serum biochemistry. In total, 43 dogs with multifocal hepatic lesions were included in this study. All dogs were classified into benign hepatic diseases (*n* = 32) and malignant haptic diseases (*n* = 11). For all dogs, their liver characteristics, lesion characteristics, and hepatobiliary ultrasound score by ultrasound were evaluated and we collected individual clinicopathological data for analysis. The findings of the univariate analysis revealed significant differences in four hematological and blood chemical parameters (hematocrit, white blood cell count, aspartate transaminase (AST), and alkaline phosphatase (ALP)) and six ultrasonographic parameters (liver parenchymal echogenicity, lesion homogeneity, lesion echogenicity, maximum lesion dimension, average lesion dimension, and hepatobiliary ultrasound score). Using multivariate analysis, only two parameters, hepatobiliary ultrasound score and lesion homogeneity, showed significant differences (*p*-value < 0.001 and *p*-value = 0.011, respectively). Additionally, these parameters demonstrated high accuracy in predicting malignant multifocal liver lesions, with accuracy rates of 97.67% and 93.02%, respectively. Therefore, the hepatobiliary ultrasound score and lesion homogeneity are considered effective parameters for screening malignant multifocal liver lesions in dogs.

## 1. Introduction

Hepatic lesions, comprising a range of abnormalities in liver tissues, including masses, tumors, cysts, and other alterations, are typically detected through imaging and microscopic examination. Hepatic lesions in dogs can be categorized into multifocal, massive, and diffuse patterns [1]. Multifocal hepatic lesions in dogs are characterized by the presence of multiple abnormalities or lesions scattered throughout the liver [2,3,4]. These lesions may exhibit variations in size, shape, and consistency. Multifocal hepatic lesions arise from various causes, including inflammatory conditions, benign processes, and malignant tumors [5,6].

Previous studies have reported that the incidence of canine primary hepatic tumors accounts for approximately 0.6–1.5% of all canine tumors [7]. Of these, hepatocellular carcinoma (HCC) is the most common hepatic tumor, accounting for 53% of all primary hepatic tumors [1,3]. It is important to highlight that 43% to 75% of canine HCCs are recognized as multifocal hepatic lesions [8,9]. However, there are other hepatic diseases, such as hepatic nodular hyperplasia, vacuolar hepatopathy, and degenerative hepatopathy [3,10]. 

Dogs with multifocal hepatic lesions can present variable and non-specific clinical signs, including abdominal distention, vomiting, diarrhea, and inappetence [3]. The clinicopathological and radiographic features of HCC are similar to those of other hepatic diseases [11]. Therefore, the diagnostic techniques to confirm specific causes of multifocal hepatic lesions are challenging. The severity of multifocal hepatic lesions depends upon pathological conditions [3,12]. Sometimes, dogs with multifocal hepatic lesions have a complex disease that prevents them from having a liver biopsy [13,14].

To achieve a definitive diagnosis, precise diagnostic tools are necessary, including additional imaging techniques such as ultrasound, computed tomography (CT) scan, magnetic resonance imaging (MRI), and pathological examination [10,15,16]. Currently, liver ultrasound is commonly used to examine liver parenchyma and identify abnormalities. This tool provides information regarding the characteristics and progression of the lesions [17]. Previous studies have focused on specific ultrasonographic parameters to predict malignant disease in dogs with focal hepatic lesions [6]. Moreover, they demonstrate a trend in high hepatobiliary ultrasound scores in malignant liver diseases, especially HCC [18]. However, there is a lack of information concerning the ultrasonographic characteristics and trend in hepatobiliary ultrasound scores in dogs with multifocal hepatic lesions. This limitation prompts an intriguing consideration for non-invasive diagnostic techniques in predicting multifocal hepatic lesions in dogs.

Therefore, this study aims to investigate the relationship between demographic data, clinical signs, clinicopathology, and ultrasonographic features for predicting malignant hepatic disease in dogs with multifocal hepatic lesions. This preliminary study is expected to be useful for obtaining a tentative diagnosis of lesions prior to treatment.

## 2. Materials and Methods

### 2.1. Animals

This retrospective and prospective study comprised 43 dogs presented at Small Animal Hospital, the Faculty of Veterinary Medicine, Chiang Mai University, between 2019 and 2023. The inclusion criteria required complete medical records, the presence of multifocal hepatic lesions observed via ultrasound, and confirmation of those lesions through histopathological examination. The research protocols were approved by the Animal Ethics Committee of the Faculty of Veterinary Medicine, Chiang Mai University (S3/2566).

### 2.2. Data Collection

Information was documented on all dogs, including breed, age, sex, concurrent diseases, hematological and blood chemical profiles, ultrasonographic findings, and histopathological results. Demographic data were analyzed to identify relationships with multifocal hepatic lesions and determine the predictive factors.

### 2.3. Ultrasonographic Examination

Multifocal hepatic lesion characteristics, such as size, echogenicity, homogeneity, and liver echogenicity and homogeneity, were evaluated by an ultrasound machine (GE health care, Milwaukee, WI, USA) with a 7–12 MHz transducer probe.

The lesion size, including its maximum and average diameter, was also recorded. The maximum lesion diameter was measured from the biggest mass/nodule in the liver. The average lesion diameter was randomly measured as more than 3–5 masses/nodular lesions from the left and right liver lobes. 

The hepatobiliary ultrasound scores were measured as mentioned previously [18] and applied to evaluate the multifocal hepatic lesions in this study. The hepatobiliary ultrasound score assessment comprised three sub-evaluations: the liver surface score, parenchyma score, and biliary score. The liver surface score was classified into three levels: sharp border (score 0), mild blunt border (score 1), and blunt border (score 2). The parenchyma score consisted of two categories: parenchyma echogenicity and liver nodularity. Parenchyma echogenicity was evaluated on a scale divided into four levels (Table 1). Nodularity was assessed using three separate scores (Table 2). The biliary score was used to evaluate three attributes: the thickness of the gallbladder wall, gall sludge, and the visibility of bile ducts, according to the scoring guidelines provided in Table 3.

After assessing each ultrasound characteristic, the scores were totaled and classified into three categories: mild (0–2), moderate (3–6), and severe (7–12). All ultrasound characteristics were studied to identify correlations with hepatic diseases and predict the presence of multifocal hepatic lesions in dogs.

### 2.4. Statistical Analyses

The IBM^®^ SPSS^®^ Statistics Program Version 25 (Armonk, NY, USA) was used for statistical analysis. The categorical data, including signalment, concurrent disease, clinical signs, liver parenchymal echogenicity, liver parenchymal homogeneity, lesion echogenicity, lesion homogeneity, and hepatobiliary ultrasound score, were analyzed for differences between benign and malignant multifocal liver lesion groups using the Chi-square test and shown in numbers and percentages. The continuous variables, including age, hematological, and blood chemical profiles, were analyzed for differences between benign and malignant multifocal liver lesion groups using the Mann–Whitney U test and shown as the mean ± standard deviation. 

The correlation between clinicopathological parameters, ultrasonographic findings, hepatobiliary ultrasound score, and multifocal hepatic lesions was described using univariate analysis. In the multivariate analysis, stepwise regression was employed to identify predictive parameters, excluding confounding and collinear factors and defining significant parameters from the univariate analysis. This was conducted to differentiate between benign and malignant multifocal liver lesions in canines. In addition, the odds ratio and 95% confidence range for each parameter were computed in the multivariate model. 

Additionally, the diagnostic performance was assessed using the significant parameters from the multivariate analysis to predict multifocal liver lesions in dogs. A *p*-value < 0.05 was considered statistically significant for all analyses. 

## 3. Results

### 3.1. Demographic Data 

A total of 43 dogs were revealed to exhibit evidence of multifocal liver lesions. The sample consisted of 25 females and 18 males with a mean age of 11.2 ± 2.56 years, ranging from 8.6 to 14.6 years. The signalment (age, sex, breed) and concurrent diseases are summarized in Table 4. 

Histopathologically, 32 multifocal hepatic lesions dogs were diagnosed with benign liver diseases (74.42%), while 11 dogs had malignant liver diseases (25.58%). The benign liver disease group consisted of hepatic degeneration (*n* = 18, 56%), cholangiohepatitis (*n* = 6, 18.75%), nodular hyperplasia (*n* = 5, 15.62%), hepatocellular adenoma (*n* = 2, 6.25%), and chronic hepatitis (*n* = 1, 3.12%). The malignant liver disease group contained dogs diagnosed with hepatocellular carcinoma (*n* = 9, 81.8%), hemangiosarcoma (*n* = 1, 9.1%), and lymphoma (*n* = 1, 9.1%).

Nineteen multifocal hepatic lesion dogs showed no clinical signs (44.18%). In the group of benign multifocal hepatic lesion dogs, 56.25% (*n* = 18) were asymptomatic, whereas 90.09% (*n* = 10) of the malignant multifocal hepatic lesion dogs presented clinical signs, including abdominal distention, inappetence, abdominal pain, and vomiting (Table 5).

Moreover, 10 clinicopathological parameters, hematocrit, white blood cell count, platelet count, aspartate transaminase (AST), alanine aminotransferase (ALT), alkaline phosphatase (ALP), blood urea nitrogen (BUN), creatinine (CRE), total protein, and albumin, were statistically analyzed (Table 6). Four parameters were found to be significantly different between the benign and malignant groups: hematocrit, white blood cell count, AST, and ALP (*p*-value = 0.003, 0.024, 0.039, and 0.039, respectively).

### 3.2. Ultrasound Examination

**Figure 1:**
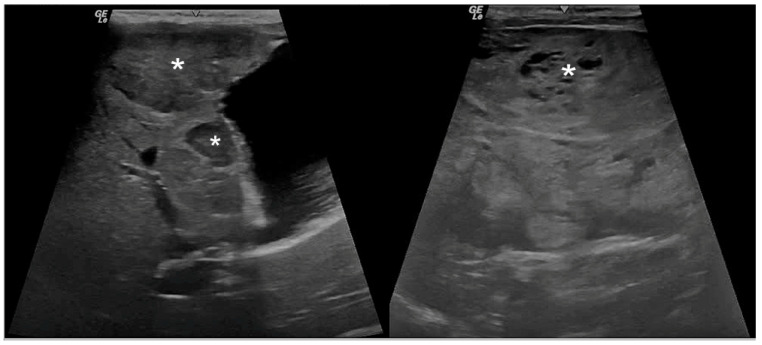
Dog. Hepatocellular carcinoma. Ultrasonographic image illustrates multifocal heterogeneous heteroechoic lesions (asterisks). Hepatobiliary ultrasound score is severe (score 7–12). B-mode (10 MH_z_ probe).

**Figure 2:**
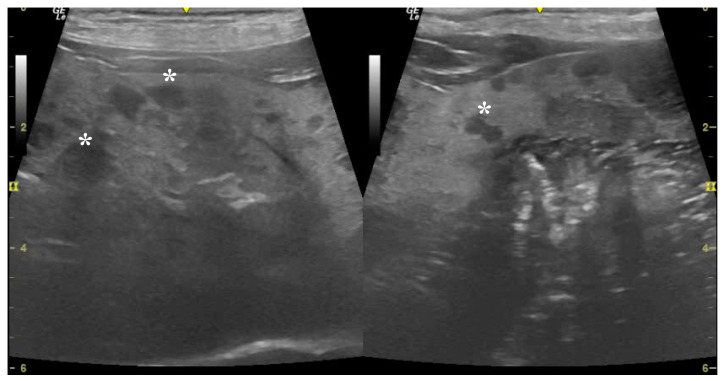
Dog. Hepatic nodular hyperplasia. Ultrasonographic image illustrates multifocal homogeneous hypoechoic lesions (asterisks) and hyperechoic liver parenchyma. Hepatobiliary ultrasound score is moderate (score 3–6). B-mode (10 MH_z_ probe).

An evaluation of seven ultrasonographic variables, four qualitative parameters, two quantitative parameters, and one semi-quantitative parameter, is summarized in Table 7. Among these, significant differences were exhibited in the ultrasound scores for liver parenchymal echogenicity, lesion homogeneity, lesion echogenicity, maximum lesion dimension, average lesion dimension, and hepatobiliary between the benign and malignant groups. Multifocal heterogeneous heteroechoic lesions with hyper- or heteroechoic liver parenchyma were common ultrasonographic characteristics of malignant groups (Figure 1). In contrast, the benign group revealed multifocal homogeneous hypoechoic lesions with hyperechoic parenchyma (Figure 2). 

**Figure 3:**
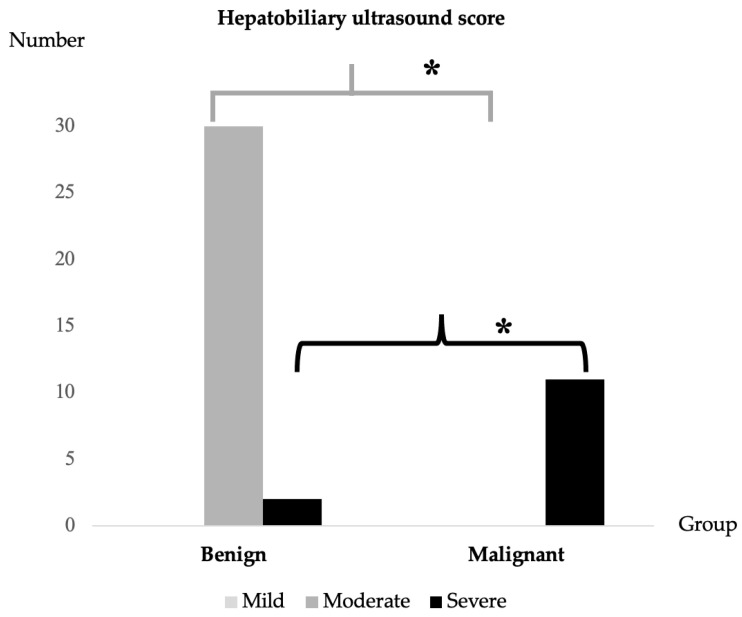
Comparison of hepatobiliary ultrasound score between benign and malignant multifocal liver lesion groups. The graph indicated that the malignant group had a severe score, whereas the majority of the benign group exhibited moderate scores, which were significantly different (* *p*-value < 0.001).

As for the hepatobiliary ultrasound score, the malignant multifocal liver lesion group showed 100% severity, whereas the benign multifocal liver lesion group had a moderate score (ranging from 3 to 5), equating to 93% (Figure 3).

All significant parameters from the univariate analysis—signalment, clinicopathology, and ultrasound evaluation—were included in the stepwise analysis. Among these parameters, the hepatobiliary ultrasound score and lesion echogenic texture were selected as candidate parameters for the multivariate analysis. The results of the multivariate analysis revealed that both parameters, namely the lesion echogenic texture (*p*-value < 0.001) and hepatobiliary ultrasound score (*p*-value = 0.011), were significantly different, enabling them to be independent variables for distinguishing benign and malignant multifocal liver lesions in dogs.

Regarding the multivariate analysis, two significant independent variables were used to determine diagnostic performance. Malignant multifocal liver lesions were found to be associated with heterogeneous lesion homogeneity (OR: 4.148, 95% CI: 0.16–0.48) and a severe hepatobiliary ultrasound score (OR: 2.669, 95% CI: 0.05–0.57) (Table 8). The diagnostic performance of heterogeneous lesion homogeneity and a severe hepatobiliary ultrasound score is shown in Table 9. The results indicated that the predictive ability of a severe hepatobiliary ultrasound score was higher than heterogeneous lesion homogeneity in predicting malignant hepatic diseases, with an area under the curve of 0.969 (Supplementary 1). The other diagnostic performance of the severe hepatobiliary score parameter showed an accuracy of 97.66%, sensitivity of 100%, and specificity of 96.85% (Table 9).

## 4. Discussion

This study is the first to identify the predictive factors of multifocal liver lesion dogs using clinicopathology, ultrasonographic characteristics, and the hepatobiliary ultrasound score. The result of the multivariate analysis revealed that only two parameters, lesion homogeneity and the hepatobiliary ultrasound score, were statically significant variables, indicating their association with malignant liver diseases.

Regarding lesion homogeneity, the heterogenous lesion echogenic texture exhibited 93.02% accuracy, 90.90% sensitivity, and 93.75% specificity in identifying malignant liver disease. The previous study mentioned that malignant liver diseases often present with a heterogeneous texture on ultrasound imaging. This appearance can also be observed in other imaging modalities such as a CT scan and MRI [19,20,21]. In a CT scan, malignant hepatic tumors often have irregular, infiltrative borders. In a CT contrast study, malignant hepatic tumors typically show heterogeneous enhancement [19,20,21]. With other modalities, such as an MRI, malignant hepatic tumors often appear heterogeneous, with high signal intensity on T2-weighted images [21]. Lesion heterogeneity is believed to be associated with fibrosis and necrosis, commonly found in malignant liver diseases [20,22]. Additionally, Cuccovillo and Lamb (2002) reported the presence of a “target sign” as a predictive characteristic on ultrasound for primary hepatic cancer in dogs, defined as an echogenic texture with a heterogeneous appearance [23,24]. These findings suggest that assessing the heterogeneity of multifocal liver lesions through imaging techniques can provide valuable information for predicting malignant liver diseases, including HCC, in dogs. However, lesion heterogeneity or target lesions can also be present in benign hepatic lesions, such as nodular hyperplasia [25]. Therefore, the accuracy and reliability of these diagnostic parameters may vary. Therefore, further research and clinical evaluations are necessary to validate their effectiveness in certain cases.

Furthermore, this study assessed the hepatobiliary ultrasound score. The results demonstrated that 100% of multifocal liver lesions associated with malignant diseases exhibited high hepatobiliary ultrasound scores (ranging from 7 to 12), whereas 93.7% of multifocal liver lesions associated with benign disease had a moderate hepatobiliary ultrasound score. Several studies have demonstrated that approximately 75% of malignant liver lesions including primary and metastatic tumors tend to have higher scores compared to benign lesions and normal liver tissues, similar to our result [18,26,27]. However, previous studies have noted that approximately 25% of malignant liver diseases may have moderate scores, potentially influenced by the stage of the disease [18,26,27]. Malignant liver diseases such as hepatocellular carcinoma, hemangiosarcoma, and lymphoma are known to exhibit rapid growth, high nodularity, and high-rated necrotic formation and induce surrounding lesion inflammation [12,28]. These features contribute to the parameters used to determine a high hepatobiliary ultrasound score. Therefore, the hepatobiliary ultrasound score may be useful for screening and predicting the severity of liver lesions, particularly in distinguishing malignant liver diseases from benign lesions and normal liver tissue. However, studies have indicated that there is no statistical difference in the hepatobiliary ultrasound score between primary and metastatic hepatic neoplasia. Therefore, using the hepatobiliary ultrasound score to differentiate the origin of the disease may have limitations [18,26,27].

Additional ultrasonographic parameters, such as the maximum and average lesion diameter, also exhibited significance in the univariate analysis. However, the maximum lesion diameter was reported as 4.33 cm, which seemed to be smaller than a previous study’s results [6]. While the lesion diameter in multifocal liver lesions may not have been extensively studied, it has been investigated in the context of focal liver lesions. Previous studies have mentioned a lesion diameter cut-off of 4.5 cm, identified by ultrasound investigation, associating it with malignant liver disease. This cut-off lesion diameter also proved significant in CT scans for predicting malignant liver diseases [1,6,19].

This study identified several significant clinicopathological parameters distinguishing between benign and malignant multifocal liver lesions. In the univariate analysis, the significant parameters were specifically revealed to be as follows: hematocrit, white blood cell count, AST, and ALP. Hematocrit is a measure of the volume percentage of red blood cells in the blood. Abnormal hematocrit levels in the context of multifocal liver lesions may be associated with underlying malignancies such as iron deficiency, hemolysis, or anemia from chronic diseases [3,29,30]. Elevated white blood cell counts may indicate infection and inflammation in the body. In the context of liver lesions, an increased white blood cell count may suggest an inflammatory response associated with a malignant lesion. This disease induces local inflammation and systemic inflammation or secondary infection [12]. In this study, elevated AST levels in the blood were found to be a significant parameter for distinguishing between benign and malignant multifocal liver lesions, indicating liver damage or injury. According to a previous study, AST is not significant because common malignant liver diseases, such as hepatocellular carcinoma, have a slow rate of hepatocyte death, resulting in the AST level being normal or mildly elevated [15]. However, some reports have indicated that certain malignant liver events, such as severe inflammation or an imbalance in neovascularization, may induce hepatocyte death and release high levels of AST into the blood [31,32]. From this study, it can be seen that 90.09% of malignant multifocal liver lesions had elevated ALP, indicating liver involvement or a biliary tract abnormality such as cholestasis [33]. In malignant liver disease, the growing mass or nodule usually compresses the biliary tract, causing bile stasis and an elevation in ALP [3].

This study demonstrates that abnormalities in hematology and blood chemistry, especially hepatobiliary enzymes, along with ultrasound characteristics and the hepatobiliary ultrasound score, can be employed to predict disease in dogs with multifocal liver lesions. Additionally, this information can be valuable for dog owners in terms of diagnosis, treatment, and prognosis. However, the limitations of this study are that variations in the number of multifocal liver lesions between groups may introduce bias, thereby affecting the interpretation of the results. Although our study results tend to differentiate between disease groups, specific diseases cannot be identified. Therefore, caution may be necessary in an assessment of these results.

## 5. Conclusions

The present study identified several factors associated with malignant multifocal liver lesions: clinical symptoms, anemia, leukocytosis, and an abundance of hepatobiliary enzymes. An association exists between malignant multifocal liver lesions and the ultrasound score. These key factors can be utilized to forecast the presence of malignant liver diseases in dogs. Furthermore, these data can be used to predict situations in which a conventional diagnosis is unattainable and may be valuable for guiding treatment decisions or facilitating a more accurate diagnosis at subsequent stages. However, in the future, additional population groups or predisposing factors may require an investigation to bolster the dependability of the data.

## Figures and Tables

**Table 1:** Parenchyma echogenicity score criteria [18].

Score	Characteristics
0	normal echogenicity
1	white (hyperechoic) or black (hypoechoic) liver parenchyma
2	parenchyma has begun to crumble but still has some normal parenchyma traits (inhomogeneous)
3	rugosity of the liver tissue (heteroechoic)

**Table 2:** Parenchyma nodularity score criteria [18].

Score	Characteristics
0	homogeneity of parenchyma and no evidence of nodular lesions
1	nodules in liver parenchyma that were distributed in less than 50% of the liver and mild nodular border of liver capsule
2	nodules in liver parenchyma that were distributed in more than 50% of the liver and diffuse nodular border of liver capsule

**Table 3:** Hepatobiliary ultrasound score criteria [18].

Score Type	Clinical Features	Score			
		0	1	2	3
Surface score Parenchymal score	Liver edge/border Parenchymal echogenicity Nodularity of parenchyma	Sharp Normal Smooth	Mild blunt Hypo-/hyper-echogenicity Mildly irregular	Blunt Inhomogeneous Irregular	Heterogeneous
Biliary score	Gallbladder wall thickness Gall sludge Bile duct visibility	<2 mm Normal No	>2 mm Increased Yes	Stellate sludge	Stone

**Table 4:** Signalment and concurrent diseases of benign and malignant multifocal liver lesion dogs.

Variables ^a^	Benign (*n* = 32)	Malignant (*n* = 11)	*p*-Value ^b^
Age (years) (mean ± SD)	11.9 ± 2.79	12.98 ± 1.58	0.302
Sex			0.668
Male (*n* (%))	14 (43.75)	4 (36.36)	
Female (*n* (%))	18 (56.25)	7 (63.64)	
Breed			0.137
Mixed (*n* (%))	10 (31.25)	3 (27.27)	
Shi-Tzu (*n* (%))	9 (28.12)	1 (9.09)	
Poodle (*n* (%))	4 (12.5)	2 (18.18)	
Chihuahua (*n* (%))	4 (12.5)	1 (9.09)	
Beagle (*n* (%))	0 (0)	2 (18.18)	
Other (*n* (%))	4 (12.5)	2 (18.18)	
Concurrent diseases			0.562
- Hyperadrenocorticism (*n* (%))	4 (12.5)	3 (27.27)	
- Atopy (*n* (%))	2 (6.25)	1 (9.09)	
- MMVD (*n* (%))	1 (3.12)	1 (9.09)	
- Neoplasia (*n* (%))	5 (15.62)	0 (0)	
- CKD (*n* (%))	1 (3.12)	0 (0)	
- No (*n* (%))	19 (59.37)	6 (54.54)	

^a^ MMVD = myxomatous mitral valve degeneration; CKD = chronic kidney disease; No = no. concurrent diseases; *n* = numbers; ^b^ *p*-value < 0.05.

**Table 5:** Clinical presentation of benign and malignant multifocal liver lesion dogs.

Clinical Sign	Benign (*n* = 32)	Malignant (*n* = 11)	*p*-Value ^a^
Asymptomatic (*n* (%))	18 (56.25)	1 (9.09)	0.007
Abdominal distention (*n* (%))	7 (21.87)	10 (90.90)	<0.001
Abdominal pain (*n* (%))	3 (9.37)	6 (54.54)	0.001
Inappetence (*n* (%))	7 (21.87)	9 (81.81)	<0.001
Vomiting (*n* (%))	2 (6.25)	4 (36.36)	0.013
Diarrhea (*n* (%))	1 (3.12)	0 (0)	0.553

^a^ *p*-value < 0.05.

**Table 6:** Clinicopathological profile of benign and malignant multifocal liver lesion dogs.

Parameter ^a^	Benign	Malignant	*p*-Value ^b^
Hematocrit (%) (mean ± SD)	46.40 (±7.66)	35.63 (±10.37)	0.003
- Normal (*n* (%))	30 (93.75)	5 (45.45)	
- Anemia (*n* (%))	2 (6.25)	6 (54.54)	
WBC (×10^3^/µL) (mean ± SD)	12.51 (±4.69)	35.12 (±26.33)	0.024
- Normal (*n* (%))	29 (90.62)	5 (45.45)	
- Leukocytosis (*n* (%))	3 (9.37)	6 (54.54)	
Platelet count (×10^3^/µL) (mean ± SD)	382.43 (±144.31)	423.18 (±244.83)	0.707
- Normal (*n* (%))	27 (84.37)	6 (54.54)	
- Thrombocytosis (*n* (%))	1 (3.12)	3 (27.27)	
- Thrombocytopenia (*n* (%))	4 (12.5)	2 (18.18)	
AST (U/L) (mean ± SD)	36.39 (±35.28)	117.21 (147.67)	0.039
- Normal (*n* (%))	24 (75)	8 (72.72)	
- Elevated ≤3 times (*n* (%))	7 (21.87)	2 (18.18)	
- Elevated ≥3 times (*n* (%))	1 (3.12)	1 (9.09)	
ALT (U/L) (mean ± SD)	216.96 (±291.13)	1019.18 (±2023)	0.095
- Normal (*n* (%))	16 (50)	4 (36.36)	
- Elevated ≤3 times (*n* (%))	10 (31.25)	1 (9.09)	
- Elevated ≥3 times (*n* (%))	6 (18.75)	6 (54.54)	
ALP (U/L) (mean ± SD)	703.65 (±787.96)	1298.45 (±773.89)	0.039
- Normal (*n* (%))	9 (28.15)	1 (9.09)	
- Elevated ≤3 times (*n* (%))	8 (25)	0 (0)	
- Elevated ≥3 times (*n* (%))	15 (46.87)	10 (90.90)	
BUN (mg/dL) (mean ± SD)	18.57 (±8.5)	28.28 (±27.65)	0.846
- Normal (*n* (%))	30 (93.75)	6 (54.54)	
- Decreased (*n* (%))	2 (6.25)	2 (18.18)	
- Increased (*n* (%))	0 (0)	3 (27.27)	
Creatinine (mg/dL) (mean ± SD)	1.023 (±0.28)	1.047 (±0.48)	0.686
- Normal (*n* (%))	32 (100)	10 (90.90)	
- Decreased (*n* (%))	0 (0)	0 (0)	
- Increased (*n* (%))	0 (0)	1 (9.09)	
Total protein (g/dL) (mean ± SD)	7.39 (±1.01)	7.06 (±0.67)	0.236
- Normal (*n* (%))	28 (87.5)	11 (100)	
- Decreased (*n* (%))	2 (6.25)	0 (0)	
- Increased (*n* (%))	2 (6.25)	0 (0)	
Albumin (g/dL) (mean ± SD)	3 (±0.3)	2.86 (±0.68)	0.878
- Normal (*n* (%))	24 (75)	6 (54.54)	
- Hypoalbuminemia (*n* (%))	8 (25)	5 (45.45)	
- Hyperalbuminemia (*n* (%))	0 (0)	0 (0)	

^a^ WBC = white blood cell count; AST = aspartate transaminase; ALT = alanine aminotransferase; ALP = alkaline phosphatase; BUN = blood urea nitrogen. ^b^ *p*-value < 0.05.

**Table 7:** The ultrasonographic parameters of multifocal liver lesion in benign and malignant groups.

Ultrasonographic Parameters	Benign *n* = 32	Malignant *n* = 11	*p*-Value ^a^
Liver parenchyma homogeneity			0.011
Homogeneous (*n* (%))	20 (62.5)	3 (27.27)	
Heterogeneous (*n* (%))	12 (37.5)	8 (72.72)	
Liver parenchyma echogenicity			0.053
Hyperechoic (*n* (%))	30 (93.75)	8 (72.72)	
Hypoechoic (*n* (%))	1 (3.12)	0 (0)	
Heteroechoic (*n* (%))	1 (3.12)	3 (27.27)	
Lesion homogeneity			<0.001
Homogeneous (*n* (%))	30 (93.75)	1 (9.09)	
Heterogeneous (*n* (%))	2 (6.25)	10 (90.90)	
Lesion echogenicity			<0.001
Hyperechoic (*n* (%))	2 (6.25)	0 (0)	
Hypoechoic (*n* (%))	29 (90.62)	3 (27.27)	
Heteroechoic (*n* (%))	1 (3.12)	8 (72.72)	
Maximum lesion diameter (cm) (mean ± SD)	1.23 (±0.68)	4.33 (±1.52)	<0.001
Average lesion diameter (cm) (mean ± SD)	0.64 (±0.42)	2.6 (±1.03)	<0.001
Hepatobiliary ultrasound score			<0.001
Mild (score 0–2) (*n* (%))	0 (0)	0 (0)	
Moderate (score 3–5) (*n* (%))	30 (93.75)	0 (0)	
Severe (score 6–12) (*n* (%))	2 (6.25)	11 (100)	

^a^ *p*-values < 0.05.

**Table 8:** Multivariate analysis of statistically significant univariable analysis parameters to distinguish malignant from benign multifocal liver lesion.

Parameters	Odds Ratio	95% CI	*p*-Value ^a^
Qualitative ultrasound parameters			
Heterogeneous lesion homogeneity	4.148	0.16–0.48	<0.001
Semiquantitative ultrasound parameter			
Severe hepatobiliary ultrasound score	2.669	0.05–0.57	0.011

^a^ *p*-values < 0.05.

**Table 9:** Diagnostic performance of independent ultrasound parameters for predicting malignant multifocal liver lesions.

	Heterogeneous Lesion Homogeneity	Severe Hepatobiliary Ultrasound Score
Area under the curve	0.923	0.969
Sensitivity (%)	90.90	100
Specificity (%)	93.75	96.85
Positive predictive value (%)	83.33	91.66
Negative predictive value (%)	96.77	100
Accuracy (%)	92.30	97.66

## Data Availability

The data are contained within the article.

## Supplementary Materials

The following supporting information can be downloaded at https://www.mdpi.com/article/10.3390/ani14192910/s1, Supplementary 1. The ROC curve of predictive factors of malignant multifocal hepatic lesions.
