# Supplementary figures and images for: Predictive Factors of Canine Malignant Hepatic Diseases with Multifocal Hepatic Lesions Using Clinicopathology, Ultrasonography, and Hepatobiliary Ultrasound Scores
Source: Animals (Basel). 2024 Oct 9;14(19):2910. doi: 10.3390/ani14192910 (PMC11476315; doi:10.3390/ani14192910)

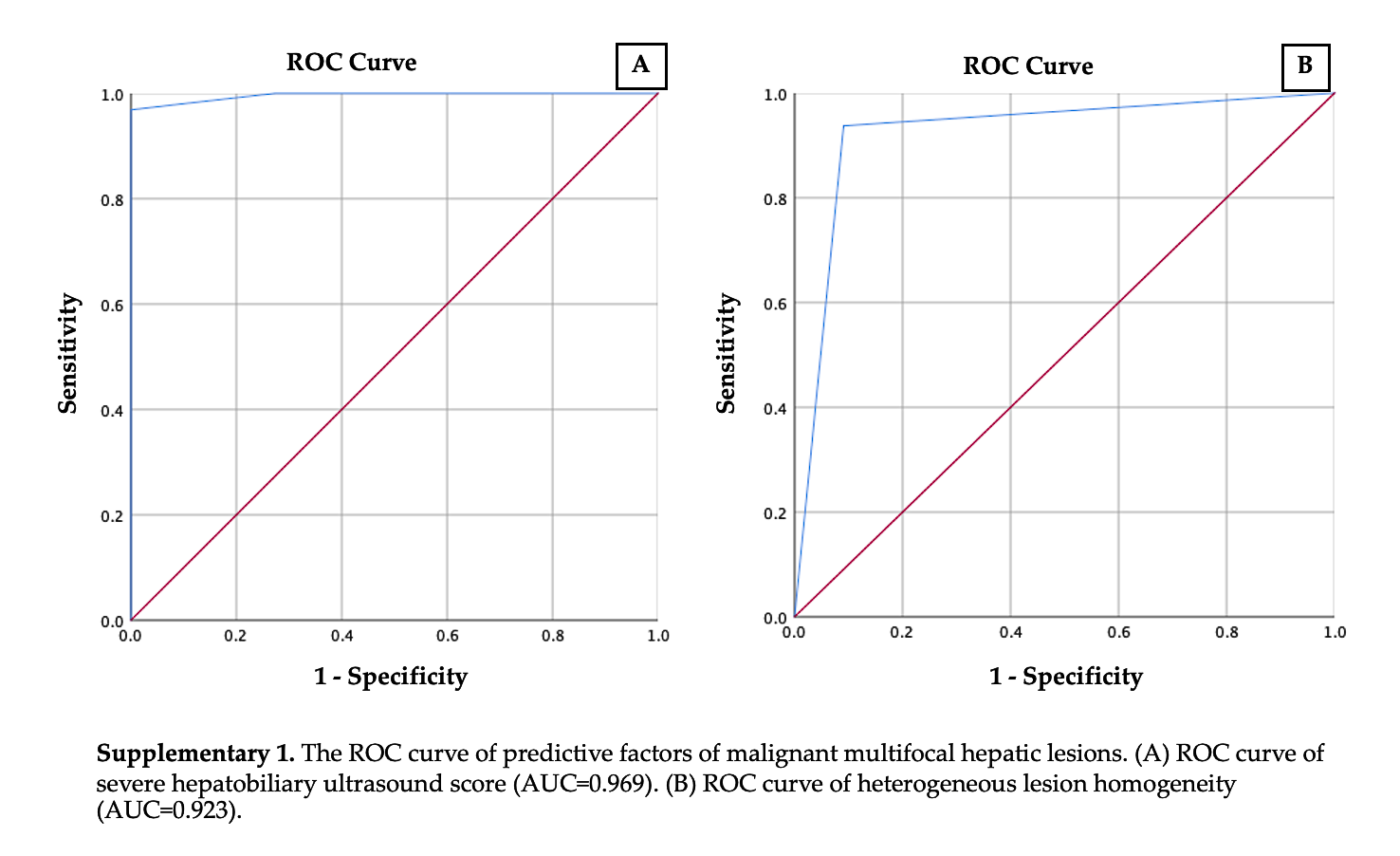

Supplement: Supplementary file 1 [file animals-14-02910-s001.zip › animals-3226592-supplementary.tif]
